# Cardiovascular mortality associated with testosterone therapy in cisgender women and transgender men: a systematic review

**DOI:** 10.3389/fendo.2026.1789504

**Published:** 2026-02-11

**Authors:** Diogo Pinto da Costa Viana, Lucas Caseri Câmara, Lucio de Sousa Monte Alto

**Affiliations:** 1 Department of Gynecology, Brazilian Society for Research and Teaching in Medicine, Florianópolis, Brazil; 2 Department of Gynecology, Brazilian Society of Obesity Medicine, Florianópolis, Brazil; 3 Department of Specialization in Clinical Anabolism, College of Governance, Engineering, and Education of São Paulo - FGE-SP, São Paulo, Brazil

**Keywords:** cardiovascular mortality, cardiovascular risk factors, cisgender women, gender-affirming hormone therapy, heart disease risk factors, testosterone therapy, transgender men

## Abstract

**Background:**

Testosterone therapy is increasingly prescribed in cisgender women for sexual and metabolic indications and constitutes the cornerstone of gender-affirming hormone therapy in transgender men. However, the nature and certainty of cardiovascular safety evidence supporting testosterone use differ across clinical contexts.

**Objective:**

To synthesize the available evidence on cardiovascular mortality associated with testosterone therapy in cisgender women and transgender men, with specific attention to study design, duration of follow up, and certainty of evidence. This review does not aim to directly compare cardiovascular risk between populations.

**Methods:**

This systematic review was conducted in accordance with the PRISMA 2020 guidelines and prospectively registered in PROSPERO (CRD420251009443). Randomized controlled trials evaluating transdermal testosterone therapy in cisgender women and observational cohort studies assessing testosterone therapy in transgender men were included. Risk of bias was assessed using the Cochrane RoB 2.0 tool and the Newcastle Ottawa Scale, and certainty of evidence was evaluated using the GRADE framework. Due to substantial clinical and methodological heterogeneity, quantitative meta-analysis was not performed.

**Results:**

Thirteen randomized controlled trials involving 2, 628 cisgender women receiving transdermal testosterone for periods ranging from 8 to 52 weeks reported no cardiovascular deaths. Thirteen observational cohort studies including 7, 837 transgender men receiving long term testosterone therapy reported 34 cardiovascular deaths, corresponding to an incidence rate of 1.81 per 1, 000 person years. The certainty of evidence was rated as moderate for short term outcomes in cisgender women and low to very low for long term cardiovascular outcomes in transgender men.

**Conclusions:**

The available evidence on cardiovascular mortality associated with testosterone therapy differs substantially between cisgender women and transgender men, primarily reflecting differences in study design, follow up duration, and certainty of evidence rather than definitive conclusions regarding comparative cardiovascular risk. The absence of cardiovascular deaths in short-term randomized trials does not allow inference regarding long term cardiovascular safety, highlighting the need for adequately powered studies with extended follow-up and standardized outcome definitions.

**Systematic review registration:**

https://www.crd.york.ac.uk/prospero/, identifier CRD420251009443.

## 1 Introduction

The therapeutic use of testosterone in women remains a subject of ongoing clinical debate, primarily due to persistent concerns about its long term safety, particularly regarding cardiovascular risk. While existing randomized clinical trials have not shown an increased incidence of adverse cardiovascular events, their limited follow up durations and the historical absence of testosterone formulations specifically approved for women continue to prompt caution among healthcare professionals and regulatory bodies (1).

Over the past two decades, clinical evidence has demonstrated that testosterone is effective in improving sexual function, particularly desire, arousal, and satisfaction, in postmenopausal cisgender women with hypoactive sexual desire disorder (HSDD). Nonetheless, international guidelines have remained conservative, due in part to the predominantly off label use of testosterone in many countries, the absence of large multicenter trials with representative samples and adequate statistical power, and the lack of standardized formulation (1, 2).

Meta-analyses of randomized clinical trials have reported changes in lipid parameters, including reductions in high-density lipoprotein cholesterol and increases in low-density lipoprotein cholesterol. However, these findings represent surrogate metabolic markers, and no consistent evidence has linked such changes to incident cardiovascular events in women (3).

Currently, testosterone is recommended solely for the treatment of HSDD in postmenopausal women. However, most supporting studies are short term, limiting the generalizability of their findings to long-term clinical use (1, 4).

In contrast, testosterone is routinely prescribed to transgender men as part of gender affirming hormone therapy, typically at doses intended to achieve male physiological serum levelsand administered for long term or lifelong use. International guidelines endorse this practice as effective and safe, provided that appropriate clinical monitoring is maintained (5, 6).

Despite reported metabolic changes, available studies in transgender men have not demonstrated a consistent increase in major cardiovascular events. However, most evidence is derived from observational cohorts with heterogeneous designs and outcome definitions.

This systematic review aims to synthesize the available evidence on cardiovascular mortality associated with testosterone therapy in cisgender women and transgender men, with specific attention to study design, duration of follow up, and certainty of evidence. This review does not aim to directly compare cardiovascular risk between populations.

Recognizing the ethical and logistical limitations of conducting randomized trials in transgender populations, this review integrates evidence from randomized controlled trials in cisgender women and observational cohort studies in transgender men to examine how cardiovascular mortality evidence has been generated within distinct clinical and methodological frameworks.

## 2 Methods

### 2.1 Study design and registration

This systematic review was conducted in accordance with the Preferred Reporting Items for Systematic Reviews and Meta Analyses (PRISMA) 2020 guidelines and was prospectively registered in the PROSPERO database (CRD420251009443).

Randomized controlled trials evaluating transdermal testosterone therapy in cisgender women and observational cohort studies evaluating testosterone therapy in transgender men were eligible for inclusion. The inclusion of distinct study designs reflects feasibility and ethical constraints across clinical contexts and was prespecified.

### 2.2 Eligibility criteria

What cardiovascular mortality and metabolic outcomes are associated with testosterone therapy in transgender men compared with low dose transdermal testosterone use in cisgender women with HSDD?

#### 2.2.1 Population

This review included studies involving two distinct populations. For cisgender women, only randomized controlled trials evaluating testosterone therapy for hypoactive sexual desire disorder (HSDD) during menopause or postmenopause were considered. For transgender men, eligible studies comprised randomized controlled trials as well as prospective or retrospective cohort studies, provided they had a minimum follow up duration of at least one year and explicitly assessed cardiovascular outcomes associated with gender affirming testosterone therapy.

Studies were excluded if they did not evaluate testosterone therapy for HSDD in cisgender women or gender affirming testosterone therapy in transgender men, had a follow up duration shorter than one year in studies involving transgender men, were case reports, editorials, commentaries, or narrative reviews, did not report cardiovascular or safety related outcomes, or were animal or *in vitro* studies assessing cardiovascular risk associated with testosterone. Although randomized studies were considered eligible for transgender men, the included evidence ultimately consisted almost entirely of observational cohort studies, reflecting feasibility and ethical constraints for long-term randomized cardiovascular safety trials in this population.

#### 2.2.2 Intervention

Studies evaluating testosterone therapy irrespective of dose, route of administration, or treatment duration were included. Both placebo controlled trials and single arm studies reporting cardiovascular safety outcomes were eligible. A predefined comparator was not required for inclusion. Studies comparing testosterone with placebo, no treatment, or reporting cardiovascular safety outcomes in a single arm design were all considered acceptable.

#### 2.2.3 Outcomes to be analyzed

The primary outcome of interest was cardiovascular mortality. Definitions and ascertainment of cardiovascular mortality varied across studies, including registry-based ICD-coded causes of death, administrative/registry linkage, adjudicated outcomes, and variably specified cardiovascular classifications. This heterogeneity in outcome definitions and ascertainment is summarized in the Supplementary Material (see **Supplementary Tables 4**, **5**). Other cardiovascular and metabolic outcomes were considered secondary and were summarized descriptively.

### 2.3 Search strategy

A comprehensive literature search was performed across four electronic databases: Cochrane Library, Embase, PubMed, and Scopus. The strategy combined both controlled vocabulary (e.g., MeSH, Emtree) and free text keywords across three predefined conceptual domains: population (cisgender women, transgender men), intervention (testosterone therapy), and outcomes (cardiovascular risk and safety).

Keywords included terms for sex and gender identity (Woman, Women, Girl, Transgender keywordss, Trans Masculine Person, Transsexual, and Two Spirit Person), as well as testosterone formulations and brand name preparations such as Androderm, AndroGel, Andropatch, Androtop, Histerone, Sustanon, Testim, Testoderm, and Testopel.

Terms related to general safety and adverse outcomes were also included. Cardiovascular-specific outcome terms were deliberately excluded to maximize search sensitivity, because cardiovascular mortality and major cardiovascular events are frequently reported as secondary safety outcomes rather than as primary endpoints in testosterone trials and observational cohorts. This approach was intended to reduce the risk of missing eligible studies in which cardiovascular outcomes were not indexed or emphasized in titles/abstracts.

All searches were conducted on March 13, 2025. The full search strategy for each database is available in **Supplementary Appendix 1**.

### 2.4 Updated search strategy

An updated literature search was conducted on January 6, 2026, using the same search strategies employed in the original systematic review conducted in March 2025. The purpose of this update was to identify studies published after the initial search covering the year 2025.

The search was carried out in the Cochrane Library, Embase, PubMed, and Scopus, applying the same descriptors, free text terms, and methodological filters as previously defined.

The updated search yielded 173 records for the cisgender women population, including 65 duplicates, and 9 records for transgender men, with 3 duplicates. Following duplicate removal and title/abstract screening, full-text assessment was performed for all potentially eligible records; no additional studies fulfilled the eligibility criteria. The most common reasons for exclusion at full-text stage were ineligible population/indication, insufficient follow-up duration (transgender men <1 year), and absence of cardiovascular or safety-related outcomes. As the updated search did not result in the inclusion of additional eligible studies, the PRISMA 2020 flow diagram remained unchanged.

#### 2.4.1 Selection of studies and data extraction

All citations retrieved through the database search were deduplicated using EndNote 21 (Clarivate Analytics) and imported into the Rayyan screening platform. Following a calibration phase with a pilot screening test, two independent reviewers screened titles and abstracts against the predefined eligibility criteria. Articles considered potentially eligible were retrieved in full and independently reassessed for final inclusion. Discrepancies between reviewers were resolved through discussion and consensus. When consensus could not be reached, a third reviewer was consulted to adjudicate.

Data extraction was independently performed by two reviewers using a standardized data extraction form developed by the authors. This process was conducted in a blinded panel based format to minimize bias. Data were independently extracted by two reviewers, using a standardized data extraction form, including information on study design, population characteristics, testosterone formulation and dosing, duration of follow up, and cardiovascular outcomes.

#### 2.4.2 Risk of bias assessment

Risk of bias was assessed using the Cochrane Risk of Bias 2.0 tool(RoB 2) (7) for randomized controlled trials and the Newcastle Ottawa Scale (NOS) (8) for observational cohort studies.

#### 2.4.3 Certainty of evidence (GRADE)

The certainty of evidence for cardiovascular mortality and secondary outcomes was assessed using the Grading of Recommendations Assessment, Development and Evaluation (GRADE) framework. GRADE assessments were performed by outcome and were not contingent on quantitative pooling.

## 3 Results

### 3.1 Study selection

A total of 1, 876 records were identified through database searches, including studies involving cisgender women (n = 1, 842) and transgender men (n = 34). After removal of duplicates and screening of titles and abstracts, full text articles were assessed for eligibility.

Following application of the predefined inclusion criteria, 26 studies were included in the final qualitative synthesis, comprising 13 randomized controlled trials in cisgender women (**Supplementary Tables 1.A, 1.B**) and 13 observational cohort studies in transgender men (**Supplementary Tables 2.A, 2.B**). The study selection process is summarized in the PRISMA 2020 flow diagram (9) (**Supplementary Figure 1**).

### 3.2 Characteristics of included studies

Among cisgender women, all included studies were randomized, placebo controlled trials evaluating low dose transdermal testosterone administered via patches, sprays, or gels. Follow up durations ranged from 8 to 52 weeks. None of the trials reported cardiovascular mortality during the study period. All trials were primarily designed to assess sexual function outcomes, with cardiovascular events reported as safety endpoints (10–22).

For transgender men, eligible studies consisted predominantly of observational cohort designs, including six retrospective cohorts, six prospective cohorts, and one randomized study. All included studies had follow-up durations of at least one year and explicitly reported cardiovascular outcomes or mortality (23–35). Follow-up periods ranged from one year to several decades.

### 3.3 Risk of bias assessment

#### 3.3.1 Cisgender women (randomized controlled trials)

Risk of bias in the 13 randomized controlled trials was assessed using the Cochrane Risk of Bias 2.0 (RoB 2) tool, with most trials judged to have low risk of bias across domains. Two trials were rated as having some concerns and one trial was classified as high risk of bias. The distribution of risk of bias judgments is presented in **Supplementary Figure 2**.

#### 3.3.2 Transgender men (observational studies)

Methodological quality of observational studies was assessed using the Newcastle–Ottawa Scale. Most studies achieved high scores in the selection domain, while comparability was limited in several studies. NOS domain scores are summarized in **Supplementary Figure 3**.

### 3.4 Synthesis of results

Due to substantial clinical, methodological, and statistical heterogeneity across studies—including differences in population (cisgender women versus transgender men), testosterone exposure context (female physiologic replacement versus masculinizing dosing), formulations and regimens, follow-up duration (weeks versus years/decades), and cardiovascular mortality ascertainment (registry-based ICD coding, adjudicated outcomes, or variably classified causes of death)—a quantitative meta-analysis was not performed. Additionally, all randomized trials in cisgender women reported zero cardiovascular deaths, and outcome definitions and ascertainment were inconsistent across cohorts, conditions under which pooled estimates may be statistically unstable and potentially misleading. Key outcomes are summarized narratively and in the Summary of Findings tables (**Supplementary Tables 3, 4**).

Formal assessments of publication bias, such as funnel plot asymmetry or Egger’s test, were not applicable due to the absence of quantitative synthesis and the limited number of cardiovascular events.

### 3.5 Outcomes for cisgender women

Thirteen randomized controlled trials comprising 2, 628 cisgender women evaluated low-dose transdermal testosterone therapy. No major adverse cardiovascular events or cardiovascular deaths were reported during follow-up periods of up to 52 weeks.

#### 3.5.1 Cardiovascular outcomes

Across all included randomized controlled trials, no major adverse cardiovascular events or cardiovascular deaths were reported in either the testosterone or placebo groups during the follow-up periods assessed. However, follow-up duration was limited (maximum 52 weeks), and the trials were not powered to detect rare outcomes such as cardiovascular mortality. No significant differences in all-cause mortality were observed. According to the GRADE framework, the certainty of evidence for cardiovascular mortality was rated as moderate and downgraded due to serious imprecision related to short follow-up duration and absence of events (**Supplementary Table 3**).

#### 3.5.2 Other clinical outcomes

Testosterone therapy was associated with improvements in sexual function outcomes and a higher frequency of mild androgenic adverse effects, such as acne and hirsutism, compared with placebo. These effects were generally described as mild and reversible across trials. According to the GRADE framework, the certainty of evidence for these outcomes ranged from moderate for sexual function outcomes to high for androgenic adverse events, and detailed results are summarized in **Supplementary Table 3**.

### 3.6 Outcomes for transgender men

Across observational cohort studies including 7, 837 transgender men, a total of 34 cardiovascular deaths were reported, corresponding to an incidence rate of 1.81 per 1, 000 person-years.

#### 3.6.1 Cardiovascular mortality

Across seven studies reporting cardiovascular mortality, a total of 34 cardiovascular deaths were observed, corresponding to an incidence rate of 1.81 deaths per 1, 000 person-years. According to the GRADE framework, the certainty of evidence for cardiovascular mortality was rated as low due to serious risk of bias inherent in observational study designs, primarily residual confounding and limited adjustment for baseline cardiovascular risk factors (**Supplementary Table 4**).

#### 3.6.2 Other health outcomes

Several studies reported changes in lipid parameters, most commonly increases in low-density lipoprotein cholesterol and decreases in high-density lipoprotein cholesterol. These findings represent surrogate metabolic markers rather than clinical cardiovascular outcomes, and the certainty of evidence for metabolic outcomes was rated as very low due to serious risk of bias and indirectness. Additionally, five observational studies reported secondary/exploratory safety outcomes related to suicide risk and found no statistically significant association with testosterone therapy (RR 1.08, 95% CI 0.81–1.45), with low certainty of evidence due to imprecision (**Supplementary Table 4**).

A descriptive overview of testosterone exposure, follow-up duration, and reported cardiovascular mortality rates across study populations is presented in **Supplementary Table 5** and illustrated in **Supplementary Figure 4**. Population-level cardiovascular mortality rates were included solely for contextual reference and are not intended for direct comparison due to differences in age distribution, outcome definitions, and follow-up duration.

### 3.7 Certainty of evidence (GRADE)

GRADE framework, the certainty of evidence was rated as moderate for short-term cardiovascular mortality outcomes in cisgender women and low to very low for long-term cardiovascular outcomes in transgender men.

## 4 Discussion

This systematic review synthesizes the available evidence on cardiovascular mortality associated with testosterone therapy in cisgender women and transgender men, highlighting how differences in study design, duration of follow-up, and certainty of evidence shape cardiovascular safety inference across clinical contexts (1, 3, 6, 36).

In cisgender women, no cardiovascular deaths were reported across randomized controlled trials evaluating transdermal testosterone therapy with follow up durations ranging from 8 to 52 weeks (10–22). This absence of reported events should be interpreted within the context of short-term exposure and limited statistical power to detect rare outcomes (1, 4), rather than as evidence of long-term cardiovascular safety (7). In transgender men, cardiovascular mortality estimates were derived exclusively from observational cohort studies with extended follow-up periods (23–35). These studies provide descriptive information on long-term outcomes under real-world conditions but do not permit causal inference. Residual confounding, differences in baseline cardiovascular risk profiles, variability in testosterone formulations and dosing regimens, and heterogeneity in outcome ascertainment limit the interpretability of these findings (6, 28, 36–38). These limitations are inherent to observational evidence and are particularly relevant when evaluating long-term safety outcomes influenced by multifactorial risk determinants.

Population-level cardiovascular mortality benchmarks were incorporated solely to contextualize the magnitude of observed rates and should not be interpreted as evidence of equivalence or difference in cardiovascular risk across populations (6, 36). Differences in age distribution, outcome definitions, follow up duration, and underlying health characteristics preclude direct comparisons between cohort derived estimates and general population mortality statistics. When interpreted alongside **Supplementary Figure 4**, the available data illustrate how cardiovascular mortality has been evaluated across markedly different exposure contexts, including short-term randomized trials in cisgender women receiving testosterone at female physiological levels, long-term observational follow-up in transgender men receiving testosterone at masculine physiological levels, and population-level reference estimates. This visual synthesis is intended to contextualize exposure duration and physiological testosterone ranges rather than to imply equivalence or difference in cardiovascular risk across populations, which cannot be established within the constraints of the available evidence.

Meta-analysis was not performed due to fundamental differences in clinical context, exposure definitions, follow-up duration, and outcome reporting across studies, as well as the presence of rare events and zero-event trials, which could have generated misleading summary estimates (9). Metabolic changes reported in transgender men, including increases in low-density lipoprotein cholesterol and decreases in high-density lipoprotein cholesterol, represent surrogate markers rather than direct clinical cardiovascular outcomes (30, 35, 37). While these findings may inform mechanistic hypotheses regarding testosterone-related metabolic effects, their prognostic significance for cardiovascular mortality remains uncertain and contributes to the overall indirectness of the available evidence.

Certainty of evidence, as assessed using the GRADE framework, further reflects differences in the underlying evidentiary base rather than definitive conclusions regarding safety (7). Moderate certainty was assigned to short-term cardiovascular mortality outcomes in cisgender women, reflecting randomized study design but constrained by short follow-up duration and imprecision (10–22). In contrast, low to very low certainty was assigned to long-term cardiovascular mortality outcomes in transgender men due to observational study design, risk of bias, imprecision, and indirectness (23–35). These ratings indicate limitations in evidence quality rather than confirmation of harm or safety.

Taken together, these findings demonstrate that cardiovascular safety evidence for testosterone therapy is shaped by differences in study design, feasibility, and duration of follow-up across clinical contexts. Short-term randomized data inform practice in cisgender women, whereas long-term testosterone exposure in transgender men is primarily evaluated through observational evidence of lower certainty (5, 6). Recognizing this distinction is essential to avoid overinterpretation of available data and to ensure that clinical and regulatory considerations are grounded in consistent evidentiary principles rather than inferred differences in risk.

Several limitations of this review should be acknowledged. Heterogeneity in study populations, exposure definitions, testosterone formulations, and outcome reporting precluded quantitative synthesis (9). Cardiovascular mortality was not a primary endpoint in most randomized trials involving cisgender women, limiting event capture and statistical power (10–22). Observational studies in transgender men were variably adjusted for traditional cardiovascular risk factors and may be influenced by unmeasured confounders, including lifestyle factors, comorbidities, psychosocial stressors, and healthcare access (6, 28). Additionally, safety findings in cisgender women cannot be extrapolated to off-label testosterone formulations or supraphysiological dosing regimens, which remain in use but lack robust cardiovascular safety data.

Despite these limitations, this review provides a structured synthesis of the available evidence on cardiovascular mortality associated with testosterone therapy and delineates key gaps in the current literature. Future research should prioritize adequately powered, long-term studies with standardized cardiovascular outcome definitions and comprehensive adjustment for baseline cardiovascular risk factors across populations receiving testosterone therapy to improve the interpretability and comparability of cardiovascular safety evidence (6, 36).

## 5 Conclusion

The available evidence on cardiovascular mortality associated with testosterone therapy differs substantially between cisgender women and transgender men, primarily reflecting differences in study design, duration of follow up, and certainty of evidence instead of establishing definitive regarding comparative cardiovascular risk. The absence of reported cardiovascular deaths in short term randomized trials involving cisgender women does not allow inference regarding long term cardiovascular safety, while cardiovascular mortality estimates derived from observational studies in transgender men remain subject to residual confounding and limited certainty.

These findings highlight important gaps in the current evidence base and underscore the need for adequately powered, long-term studies with standardized cardiovascular outcome definitions and comprehensive adjustment for baseline cardiovascular risk factors. Addressing these gaps is essential to improve the interpretability and comparability of cardiovascular safety evidence across populations receiving testosterone therapy.

## Data Availability

The original contributions presented in the study are included in the article/**Supplementary Material**. Further inquiries can be directed to the corresponding author.

## Supplementary material

Supplementary Figure S4 Visual Summary of Cardiovascular Mortality Outcomes Relative to Duration of Testosterone Exposure in the Included Studies. This figure illustrates the critical disparity in study duration and evidence types between populations. The top panel shows cisgender women receiving transdermal testosterone in RCTs. While zero cardiovascular deaths were reported (N=2,628), these trials were restricted to short-term follow-up (maximum 52 weeks). The bottom panel shows transgender men receiving masculinizing hormone therapy in observational cohorts, indicating a cardiovascular mortality rate of 1.81 deaths per 1,000 person-years over a significantly longer mean follow-up of 5.7 years (N=16,242). Important Interpretation Note: The juxtaposition of these distinct populations is intended solely to highlight the gap in long-term data for cisgender women versus transgender men. The absence of observed cardiovascular events in the cisgender women group reflects insufficient statistical power and short exposure duration to detect rare, long-latency events, and must not be interpreted as confirmation of long-term cardiovascular safety. Direct comparison of risk rates between these groups is methodologically invalid due to fundamental heterogeneity in study designs (RCT vs. Observational), baseline population characteristics, routes of administration (transdermal stable levels vs. varied formulations including intramuscular peaks), and vastly different exposure timelines. CV = cardiovascular; RCT = randomized controlled trial.
