# Supplementary material for: Cardiovascular mortality associated with testosterone therapy in cisgender women and transgender men: a systematic review
Source: Front Endocrinol (Lausanne). 2026 Feb 11;17:1789504. doi: 10.3389/fendo.2026.1789504 (PMC12932213; doi:10.3389/fendo.2026.1789504)
Supplement: Supplementary Figure S4 — Visual Summary of Cardiovascular Mortality Outcomes Relative to Duration of Testosterone Exposure in the Included Studies. This figure illustrates the critical disparity in study duration and evidence types between populations. The top panel shows cisgender women receiving transdermal testosterone in RCTs. While zero cardiovascular deaths were reported (N=2,628), these trials were restricted to short-term follow-up (maximum 52 weeks). The bottom panel shows transgender men receiving masculinizing hormone therapy in observational cohorts, indicating a cardiovascular mortality rate of 1.81 deaths per 1,000 person-years over a significantly longer mean follow-up of 5.7 years (N=16,242). Important Interpretation Note: The juxtaposition of these distinct populations is intended solely to highlight the gap in long-term data for cisgender women versus transgender men. The absence of observed cardiovascular events in the cisgender women group reflects insufficient statistical power and short exposure duration to detect rare, long-latency events, and must not be interpreted as confirmation of long-term cardiovascular safety. Direct comparison of risk rates between these groups is methodologically invalid due to fundamental heterogeneity in study designs (RCT vs. Observational), baseline population characteristics, routes of administration (transdermal stable levels vs. varied formulations including intramuscular peaks), and vastly different exposure timelines. CV = cardiovascular; RCT = randomized controlled trial. [file DataSheet1.pdf]

Supplementary Material

Supplementary Online Content: Viana et al. Cardiovascular Risk of Testosterone Therapy in Cisgender Women and Transgender Men: A Systematic Review

Supplementary note (contextual references): References to cisgender male population data are provided solely for contextual interpretation and are not intended as formal comparators within the systematic review framework. Any visual or tabular alignment across groups is descriptive and does not support inference of equivalence or difference in cardiovascular risk across populations.

eAppendix 1: table 1. Search strategy

Population	Search strategy	Nº
Cochrane Library<br><br>**Cisgender Women**	ID Search Hits<br><br>#1 MeSH descriptor: [Women] explode all trees 1450<br><br>#2 Women Girl OR Girls OR Woman OR female 1053709<br><br>#3 MeSH descriptor: [Testosterone] explode all trees 3799<br><br>#4 Testosterone OR Androderm OR AndroGel OR Andropatch OR Androtop OR Histerone OR Sterotate OR Sustanon OR Testim OR Testoderm OR Testolin OR Testopel 9598<br><br>#5 MeSH descriptor: [Safety] explode all trees 4999<br><br>#6 Safety OR Safeties 337440<br><br>#7 #1 OR #2 1053709<br><br>#8 #3 OR #4 9658<br><br>#9 #5 OR #6 337445<br><br>#10 #7 AND #8 AND #9 575<br><br>#11 MeSH descriptor: [Clinical Study] explode all trees 42<br><br>#12 MeSH descriptor: [Clinical Trial] explode all trees 42<br><br>#13 "Randomized Controlled Trial" OR "Controlled Clinical Trial" OR Randomization OR "Random Allocation" OR "Double-Blind Method" OR "Double Blind Procedure" OR "Double-Blind Studies" OR "Single-Blind Method" OR "Single Blind Procedure" OR "Single-Blind Studies" OR Placebo* OR "Control Groups" OR "Control Group 1288098	499

	#14 #11 OR #12 OR #13 1288102<br><br>#15 #10 AND #14 499	
Cochrane Library<br><br>**Transgender Men**	ID Search Hits<br><br>#1 MeSH descriptor: [Transgender Persons] explode all trees 176<br><br>#2 "Transgender Persons" OR "Trans Masculine Person" OR "Trans-Masculine Person" OR "Trans-Masculine Person" OR transexual* OR "transmembrane Person" OR transgender* OR "Transgender Person" OR "Transgendered Person" OR "Transgendered Persons" OR "masculine person" OR "masculine persons" OR "transsexual person" OR "Two Spirit Person" OR "Two-Spirit Person" 909<br><br>#3 #1 OR #2 909<br><br>#4 MeSH descriptor: [Testosterone] explode all trees 3818<br><br>#5 Testosterone OR Androderm OR AndroGel OR Andropatch OR Androtop OR Histerone OR Sterotate OR Sustanon OR Testim OR Testoderm OR Testolin OR Testopel 9665<br><br>#6 #4 OR #5 9729<br><br>#7 MeSH descriptor: [Safety] explode all trees 5002<br><br>#8 Safety OR Safeties 341216<br><br>#9 #7 OR #8 341221<br><br>#10 MeSH descriptor: [Clinical Study] explode all trees 42<br><br>#11 "Randomized Controlled Trial" OR "Controlled Clinical Trial" OR Randomization OR "Random Allocation" OR "Double-Blind Method" OR "Double Blind Procedure" OR "Double-Blind Studies" OR "Single-Blind Method" OR "Single Blind Procedure" OR "Single-Blind Studies" OR Placebo* OR "Control Groups" OR "Control Group" 1300429<br><br>#12 MeSH descriptor: [Clinical Trial] explode all trees 42<br><br>#13 #10 OR #11 OR #12 1300433<br><br>#14 #3 AND #6 AND #9 AND #13 9	9
Embase<br><br>**Cisgender Women**	('female'/exp OR girl* OR woman OR women) AND ('testosterone'/exp OR testosterone OR androderm OR androgel OR andropatch OR androtop OR histerone OR sterotate OR sustanon OR testim OR testoderm OR testolin OR testopel) AND ('safety'/exp OR safety OR safeties) AND ('randomized controlled trial (topic)'/exp OR 'randomized controlled trial' OR 'controlled clinical trial' OR randomization OR 'random allocation' OR	371

	'double-blind method' OR 'double blind procedure' OR 'double-blind studies' OR 'single-blind method' OR 'single blind procedure' OR 'single-blind studies' OR placebo* OR 'control groups' OR 'control group')	
**Embase**<br><br>**Transgender Men**	('transgender'/exp OR 'transgender persons' OR 'trans masculine person' OR 'trans-masculine person' OR transexual* OR 'transmembrane person' OR transgender* OR 'transgender person' OR 'transgendered person' OR 'transgendered persons' OR 'masculine person' OR 'masculine persons' OR transsexual or ‘person two spirit person' or 'two-spirit person') AND ('testosterone'/exp OR testosterone OR androderm OR androgel OR andropatch OR androtop OR histerone OR sterotate OR sustanon OR testim OR testoderm OR testolin OR testopel) AND ('safety'/exp OR safety OR safeties) AND ('randomized controlled trial (topic)'/exp OR 'randomized controlled trial' OR 'controlled clinical trial' OR randomization OR 'random allocation' OR 'double-blind method' OR 'double blind procedure' OR 'double-blind studies' OR 'single-blind method' OR 'single blind procedure' OR 'single-blind studies' OR placebo* OR 'control groups' OR 'control group')	7
Medline/<br>Pubmed<br><br>**Cisgender Women**	(Woman[MH] OR Women OR Girl OR Girls) AND (Testosterone[Supplementary Concept] OR Androderm OR AndroGel OR Andropatch OR Androtop OR Histerone OR Sterotate OR Sustanon OR Testim OR Testoderm OR Testolin OR Testopel OR Testosterone) AND (Safety[MH] OR Safeties OR Safety) AND ("Randomized Controlled Trial"OR "Controlled Clinical Trial" OR randomization OR "Random Allocation"[MH] OR "Double-Blind Method" OR "Double Blind Procedure" OR "Double-Blind Studies" OR "Single-Blind Method" OR "Single Blind Procedure" OR "Single-Blind Studies" OR placebo* OR "Control Groups" OR "Control Group")	230
Medline/<br>Pubmed<br><br>**Transgender Men**	("Transgender Persons"[MH] OR Trans Masculine Person* OR Trans-Feminine Person* OR Trans-Masculine Person* OR Trans-Masculine Person* OR Transexual* OR Transfeminine Person* OR Transgender* OR "Transgender Person" OR "Transgendered Person" OR "Transgendered Persons" OR "Transmasculine Person" OR "Transmasculine Persons" OR Transsexual Person* OR Two Spirit Person* OR Two-Spirit Person*) AND (Testosterone[Supplementary Concept] OR Testosterone OR Androderm OR AndroGel OR Andropatch OR Androtop OR Histerone OR Sterotate OR Sustanon OR Testim OR Testoderm OR Testolin OR Testopel) AND (Safety[MH] OR Safeties OR Safety) AND ("Randomized Controlled Trial"OR "Controlled Clinical Trial" OR randomization OR "Random Allocation"[MH] OR "Double-Blind Method" OR "Double Blind Procedure" OR "Double-Blind Studies" OR "Single-Blind Method" OR "Single Blind Procedure" OR "Single-Blind Studies" OR placebo* OR "Control Groups" OR "Control Group")	9
Scopus	(TITLE-ABS-KEY (women OR girl* OR woman OR female) AND TITLE-ABS-KEY (testosterone OR androderm OR androgel OR andropatch OR androtop OR histerone OR sterotate OR sustanon OR	470

**Cisgender Women**	testim OR testoderm OR testolin OR testopel) AND TITLE-ABS-KEY (safety OR safeties) AND TITLE-ABS-KEY ("Randomized Controlled Trial" OR "Controlled Clinical Trial" OR randomization OR "Random Allocation" OR "Double-Blind Method" OR "Double Blind Procedure" OR "Double-Blind Studies" OR "Single-Blind Method" OR "Single Blind Procedure" OR "Single-Blind Studies" OR placebo* OR "Control Groups" OR "Control Group"))	
**Scopus**<br><br>**Transgender Men**	TITLE-ABS-KEY ("Transgender Persons" OR "Trans Feminine Person" OR "Trans Masculine Person" OR "Trans-Feminine Person" OR "Trans-Masculine Person" OR "Trans-Masculine Person" OR transexual* OR "transmembrane Person" OR transgender* OR "Transgender Person" OR "Transgendered Person" OR "Transgendered Persons" OR "masculine person" OR "masculine persons" OR transsexual AND person* OR "Two Spirit Person" OR "Two-Spirit Person") AND TITLE-ABS-KEY (testosterone OR endoderm OR androgen OR endopatch OR andrology OR ethisterone OR cerotate OR sustanon OR testis OR ectoderm OR testilin OR estoppel) AND TITLE-ABS-KEY (safety OR safeties) AND TITLE-ABS-KEY ("Randomized Controlled Trial" OR "Controlled Clinical Trial" OR randomization OR "Random Allocation" OR "Double-Blind Method" OR "Double Blind Procedure" OR "Double-Blind Studies" OR "Single-Blind Method" OR "Single Blind Procedure" OR "Single-Blind Studies" OR placebo* OR "Control Groups" OR "Control Group")	9

Appendix 2: supplementary eFigure 1. PRISMA 2020 flow diagram.

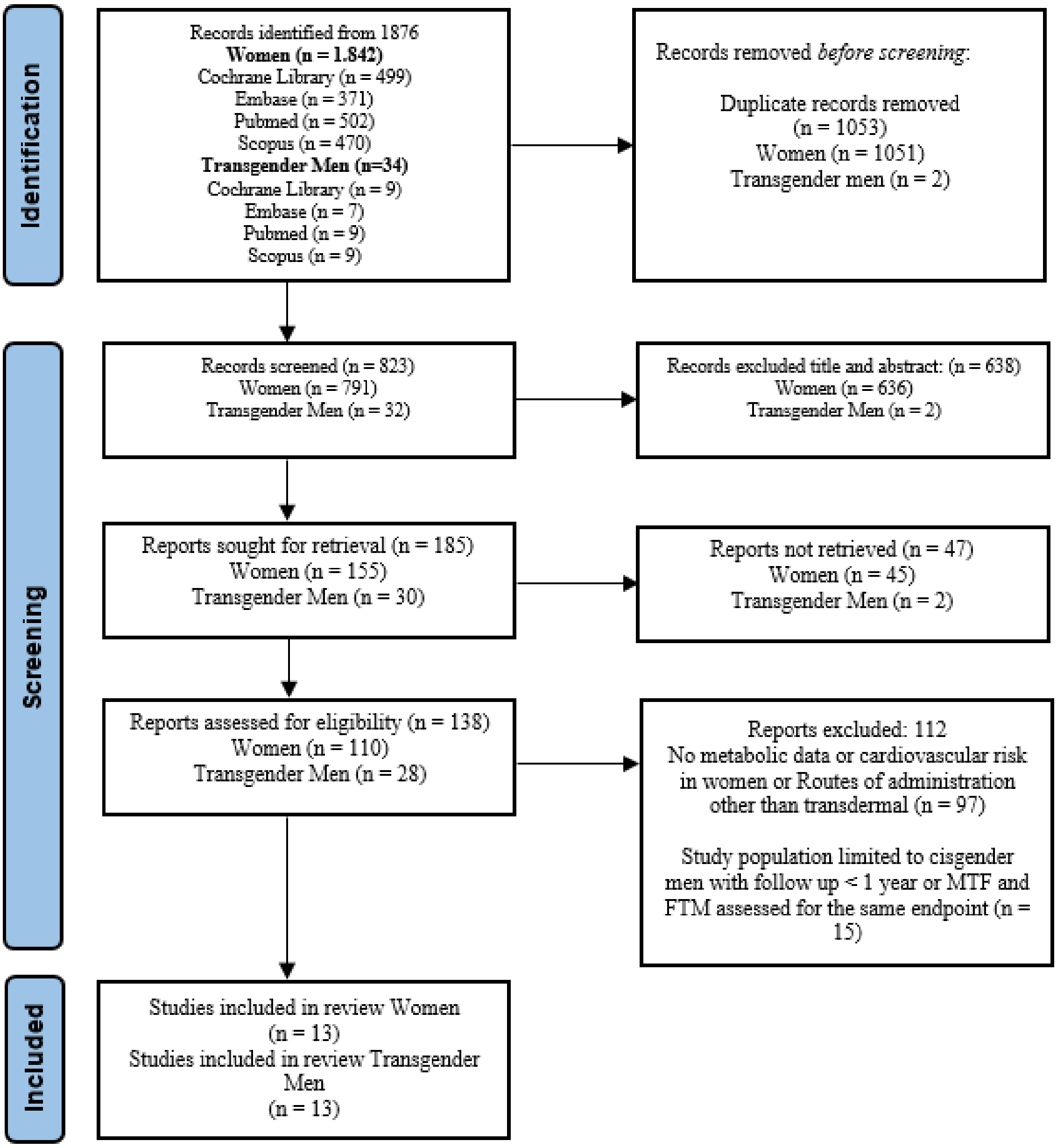

Appendix 3: Summary of the 13 Included RCTs.

eTable 1.A: Summary of the 13 Included Studies Investigating Testosterone Therapy in Cisgender Women (13–25).

Author, Year	Study Design	Sample Size	Population/Mean Age	Intervention/Dose/Frequency/Route	Follow-up Duration
Shifren et al., 2000(13)	Randomized, double-blind, placebo-controlled crossover clinical trial.	75 women<br>Intention-to-treat analysis: 65 women completed at least one treatment period.	Cisgender women aged 31–56 who had bilateral oophorectomy and hysterectomy<br>Mean age: 47 years (aged 31 to 56)	Testosterone therapy: Transdermal patches (2x/week)<br>Dosages: 150 µg/day<br>300 µg/day<br>Compared against placebo<br>All groups continued oral estrogen therapy throughout the study.	12 week
Goldstat et al., 2003(14)	Randomized, double-blind, placebo-controlled crossover trial.	Screened: 61 women<br>Randomized: 49 Completed per protocol: 34 Included in final analysis: 31 with full data.	Healthy, eugonadal premenopausal women aged 30–45 - Mean age: 39.7 years.	Testosterone therapy: 10 mg/day transdermal testosterone cream Design: Two 12-week treatment periods (testosterone and placebo), separated by a 4-week washout<br>Application: Daily on the thigh	28 weeks

Braunstein et al., 2005(15)	Randomized, placebo-controlled clinical trial	A total of 447 surgically menopausal women were randomized.<br>318 participants completed the 24-week follow-up period.	Cisgender women who had undergone surgical menopause (hysterectomy and bilateral oophorectomy) and were on stable oral estrogen therapy.<br>Age range: 24 to 70 years.	Participants received a transdermal testosterone patch, applied twice weekly to the abdomen, with doses of: 150 µg/day, 300 µg/day, 450 µg/day<br>A placebo patch group served as control.	24 weeks
Buster et al., 2005(16)	Multicenter, randomized, double-blind, placebo-controlled trial	533 women<br>266 in placebo group 267 in testosterone patch group Completed study: 417 (78%)	Surgically menopausal women (hysterectomy + bilateral oophorectomy) Mean age: 49.5 years (placebo), 48.3 years (testosterone group).	Testosterone patch (matrix design)<br>Dose: 300 µg/day<br>Route: Transdermal (applied twice weekly to the abdomen)<br>Comparator: Identical placebo patch.	Pretreatment phase: 8 weeks<br>Treatment period: 24 weeks<br>Total per-patient duration: 32 weeks.
Simon et al., 2005(17)	Randomized, double-blind, placebo-controlled clinical trial (Phase III)	562 surgically menopausal women were randomized: Placebo group: 279<br>Testosterone patch group: 283	Surgically menopausal women (post bilateral oophorectomy and hysterectomy), aged 26–70 years, diagnosed with Hypoactive Sexual Desire Disorder (HSDD) and receiving stable estrogen therapy.<br>Mean age: ~49 years.	Testosterone transdermal patch, applied twice weekly delivering 300 µg/day<br>Compared to visually identical placebo patches<br>Participants were also on stable oral or transdermal estrogen therapy.	24 weeks

Davis et al., 2006(18)	Randomized, double-blind, placebo-controlled trial	77 women Completed 24 weeks: 61 (79%)<br>Analyzed for efficacy: All participants who received ≥1 application (intent-to-treat)	Surgically menopausal women (bilateral oophorectomy + hysterectomy)<br>Age: 20–70 years (mean ~50 years)	Transdermal testosterone matrix patch (Watson Labs)<br>Dose: 300 µg/day<br>Application: Twice weekly to the abdomen<br>Comparator: Placebo patches identical in appearance	24 weeks
Nathorst-Boos et al., 2006(19)	Randomized, double-blind, placebo-controlled crossover trial	60 postmenopausal women<br>Completed and analyzed: 53 participants	Healthy postmenopausal women (50–65 years) on stable hormone replacement therapy (HRT) with decreased libido<br>Mean age: 55.4 years	Testosterone intervention: 10 mg/day transdermal testosterone gel (Testogel, Besins-Iscovesco)<br>Route: Percutaneous application on the thigh<br>Frequency: Once daily<br>Comparator: Matching placebo gel<br>Design: Each participant received both treatments for 3 months (crossover design)	24 weeks
Shifren et al., 2006(20)	Multicenter, randomized, double-blind, placebo-controlled, parallel-group trial	549 naturally menopausal women<br>Analysis population (with SHBG ≤160	Naturally menopausal women aged 40–70 years<br>Mean age: ~54 years	Transdermal testosterone patch<br>Dose: 300 µg/day<br>Frequency: Applied twice weekly<br>Route: Transdermal (abdominal)<br>All participants continued oral estrogen therapy	24 weeks

		nmol/L): 483 (88%)<br>Completed the study: 433 (79%)			
El-Hage, Eden, Manga, 2007(21)	Double-blind, randomized, placebo-controlled, crossover clinical trial	Screened: 52 women<br>Enrolled: 36<br>Completed for analysis (intention-to-treat): 36	Healthy postmenopausal hysterectomized women Condition: Hypoactive Sexual Desire Disorder (HSDD)<br>All were on transdermal estrogen therapy<br>All were in stable relationships, non-depressed, and screened for sexual dysfunction by a psychologist<br>Mean 54 years	10 mg/day testosterone cream (2 cm Andro-Feme1)<br>Route: Topical application to the forearm<br>Frequency: Once daily for 12 weeks<br>Comparator: Identical placebo cream	12 weeks
Davis et al., 2008(22)	Randomized, double-blind, placebo-controlled, dose-ranging clinical trial	261 premenopausal women	Premenopausal women aged 35–46 years with reduced sexual satisfaction and low free testosterone (<3.8 pmol/L)	Testosterone administered via a metered-dose transdermal spray (Abdomen, once daily):<br>Low dose: 56 µL/day<br>Intermediate dose: 90 µL/day<br>High dose: 2 × 90 µL/day<br>Placebo group matched in volume	16 weeks

Davis et al., 2008(23)	Randomized, double-blind, placebo-controlled clinical trial	814 postmenopausal women were randomized.<br>Completion at 24 weeks: ~71%<br>Completion at 52 weeks: ~57%	Postmenopausal women (natural or surgical menopause) with hypoactive sexual desire disorder (HSDD).<br>Age range: 40–70 years	Transdermal testosterone patch (Intrinsa)<br>Dosages: 150 µg/day, 300 µg/day<br>Frequency: Applied twice weekly (two patches, abdomen)<br>Route: Transdermal	24 weeks<br>Safety follow-up: 52 weeks<br>Extension for subset: up to 104 weeks
Panay, N. and colleagues, 2010(24)	Randomized, double-blind, placebo-controlled, multicenter clinical trial	272 naturally menopausal women were randomized: 130 received testosterone 142 received placebo	Naturally menopausal cisgender women Ages 40–70 (mean ~56.6 years)	Transdermal testosterone patch (TTP)<br>Dose: 300 µg/day<br>Frequency: Applied twice weekly<br>Route: Transdermal (abdominal patch)	24 weeks
Fooladi et al., 2014(25)	Randomized, double-blind, placebo-controlled trial	44 women randomized<br>22 received transdermal testosterone (TT)<br>22 received placebo	Cisgender women, ages 35–55<br>On stable doses of SSRIs or SNRIs for at least 3 months<br>All experienced treatment-emergent loss of libido<br>Cisgender women<br>Mean age: 47.6 years	Transdermal testosterone patch (delivered 300 mcg/day)<br>Applied twice weekly to the abdomen<br>Compared with identical placebo patch	12 weeks

eTable 1.B: Summary of the 13 Included Studies Investigating Testosterone Therapy in Cisgender Women (13–25).

**Author, Year**	**Cardiovascular Outcomes**	**Metabolic Outcomes**	**Mortality**
Shifren et al., 2000(13)	No major cardiovascular events reported	No significant changes in total cholesterol, HDL, LDL, or triglycerides across treatment arms<br>No significant changes in hematocrit	None<br>Adverse Events: Mild events led to 4 withdrawals (e.g., anxiety, nipple discharge, skin irritation)
Goldstat et al., 2003(14)	No cardiovascular events reported<br>Blood pressure: Uncontrolled hypertension was an exclusion criterion<br>Safety: No acne, voice changes, or hirsutism were observed	No change in metabolic profile	None reported<br>Serious adverse events: None occurred<br>Adherence: Well tolerated; no major withdrawals due to side effects
Braunstein et al., 2005(15)	No cardiovascular events (e.g., myocardial infarction, stroke, severe hypertension) were reported during the trial.<br>Cardiovascular Risk<br>There was no observed increase in cardiovascular risk associated with any of the testosterone doses.	There were no significant changes in lipid values or liver enzymes.<br>As expected, testosterone levels increased dose-dependently.<br>The treatment was generally well tolerated.	No deaths occurred during the study.<br>No serious adverse events were attributed to testosterone treatment.

Buster et al., 2005(16)	Adverse cardiovascular events:<br>None were attributed to the treatment<br>5 serious events in testosterone group (e.g., cellulitis, vertigo), all unrelated	No meaningful changes in:<br>Blood pressure<br>Lipid profiles<br>Liver or renal function	None<br>Serious Adverse Events: Occurred in 1.9% of testosterone group; none were drug-related<br>Androgenic AEs: Higher in testosterone group (e.g., acne, alopecia, mild voice deepening), mostly mild
Simon et al., 2005(17)	No clinically relevant changes in vital signs<br>No increase in serious cardiovascular adverse events<br>Two events in the testosterone group (1 transient ischemic attack; 1 with increased heart rate and nausea), but both continued treatment and events resolved<br>No significant difference in CV adverse event rates between groups	No clinically meaningful changes in:<br>Lipid profile<br>Glucose metabolism<br>Liver or kidney function	No deaths reported<br>No evidence that testosterone use was associated with life-threatening events
Davis et al., 2006(18)	Serious cardiovascular events: None reported	Vitals and lab parameters (lipids, glucose, coagulation): No significant changes observed<br>Blood pressure and liver enzymes: Unchanged from baseline	None<br>Serious adverse events: None<br>Mild androgenic effects: Slight hirsutism increase; one case of reversible mild voice deepening

Nathorst-Boos et al., 2006(19)	No significant changes in cardiovascular markers between testosterone and placebo phases	No significant changes in total cholesterol, HDL, LDL, or triglycerides<br>Hemoglobin and erythropoietin remained unchanged<br>Side effects: Mild androgenic effects (facial hair, acne) were similar between testosterone and placebo	None reported<br>Severe adverse events: None; one case of uterine carcinoma diagnosed early and deemed preexisting
Shifren et al., 2006(20)	No cardiovascular events were reported as related to testosterone	No clinically relevant changes in: Lipid profiles, Glucose/carbohydrate metabolism and Liver or renal function.No significant changes in blood pressure, weight, or heart rate<br>Vital signs and general labs remained stable	Two deaths occurred (both in motor vehicle accidents, unrelated to treatment)
El-Hage, Eden, Manga, 2007(21)	Monitored: Blood pressure, pulse rate, weight<br>No significant changes in cardiovascular parameters during treatment	No significant changes in cholesterol, lipids, or mood scores<br>No significant side effects like hirsutism or acne were observed<br>Weight and BMI remained stable	No deaths reported<br>No serious adverse events occurred
Davis et al., 2008(22)	No serious cardiovascular events reported<br>No increase in cardiovascular risk detected over the short	No significant changes in lipid profile, glucose, CRP, or weight	No deaths reported; one pregnancy occurred in the placebo group, leading to healthy full-term delivery

Davis et al., 2008(23)	No significant cardiovascular events were reported<br><br>Vital signs and weight remained stable.<br><br>No increase in hypertension or major adverse cardiovascular events (MACE) was observed.	No significant changes in lipids, glucose, or liver enzymes across any group	No deaths reported due to testosterone use<br><br>Breast cancer developed in 4 women (all in testosterone groups), but causality remains uncertain.<br><br>One woman had symptoms prior to randomization<br><br>One case emerged 3 months after therapy ended
Panay, N. and colleagues, 2010(24)	No myocardial infarction, stroke, or serious cardiovascular events were reported<br><br>Blood pressure and other vital signs remained stable<br><br>No evidence of increased cardiovascular risk	No significant changes in: Lipid/lipoprotein profiles Glucose or insulin metabolism Liver or kidney function	No deaths were reported<br><br>No serious safety signals related to testosterone use
Fooladi et al., 2014(25)	No myocardial infarction, stroke, or clinically significant blood pressure changes reported<br><br>No serious cardiovascular events observed	No major changes in other metabolic markers were reported<br><br>No androgenic side effects like hirsutism or acne were seen	No deaths reported<br><br>One hospitalization for depression occurred in TT group but was deemed unrelated to treatment

Appendix 4: Summary of the 13 Included cohort studies.

eTable 2.A: Summary of the 13 Included Studies Investigating Testosterone Therapy in Transgender Men (26–38).

**Author, Year**	**Study Design**	**Country**	**Sample Size**	**Population/Mean Age**	**Intervention/Dose/Frequency/Route**	**Exposure/ Follow-up Duration**
Gooren, Giltay and Bunck, 2008(26)	Observational cohort study	Amsterdam, Netherlands	3,112<br>Male-to-female (MtF): 2,236<br>Female-to-male (FtM): 876	Transgender mens FtM undergoing sex reassignment. Mean 27,3 years	FtM:<br>Testosterone esters: 250 mg intramuscular (IM) every 2 weeks<br>Some used testosterone undecanoate (1,000 mg every 10–12 weeks)<br>Transdermal gel or patches also mentioned	31 years of cumulative observation (1975–2006)
Meriggiola et. al.., 2008(27)	Prospective clinical trial (randomized comparative)	Bologna, Italy	21 trans men<br>TU alone (n=7)<br>TU + Letrozole (n=7)<br>TU + Dutasteride (n=7)	Transgender men (FtM) ~30 years	Testosterone undecanoate (TU), intramuscular (IM)<br>Dosage: 1,000 mg IM every 12 weeks (Nebido®)<br>Additional meds:<br>Group 2: Letrozole 2.5 mg/day orally<br>Group 3: Dutasteride 0.5 mg/day orally	1 year (54 weeks)
Asscheman et al., 2011(28)	Retrospective cohort study	Amsterdam, Netherlands	1,331	Transgender men - FtM: mean 26.1 years	FtM Treatment:<br>Testosterone esters: 250 mg IM every 2–3 weeks	FtM: 18.8 years

			Male-to-female (MtF): 966<br>Female-to-male (FtM): 365		Oral testosterone undecanoate (160–240 mg/day)<br>Transdermal testosterone (50 mg/day)	
Pelusi et al., 2014(29)	Prospective observational study	Bologna, Italy	45 Female-to-male (FtM) transgender<br>Divided equally into 3 groups (15 each) based on the testosterone formulation used	Female-to-male (FtM) transgender individuals<br>Mean Age: TD: 30.9 years, T-gel: 29.4, TU: 28.2	TD (Testoviron depot): IM injection 100 mg every 10 days<br>T-gel: 50 mg/day applied transdermally<br>TU (Testosterone undecanoate): IM 1,000 mg every 6 weeks initially, then every 12 weeks	1 year (54 weeks)
Wierckx et al., 2014(30)	Prospective, multicenter observational cohort study	Belgium, Norway, and the Netherlands	111 trans men and 53 trans women	Trans men (female-to-male) - 26–29 years	Trans men: Testosterone undecanoate 1000 mg IM every 12 weeks (majority)<br>Some used transdermal or oral formulations<br>Trans women: Estrogen (oral or transdermal) ± anti-androgens (cyproterone acetate)	1 year (54 weeks)
Getahun et al., 2018(31)	Retrospective cohort study	California and Georgia	4,960 Transfeminine: 2,842	Transfeminine	Intervention: Cross-hormone therapy	4 years

			Transmasculinos: 2.118	Transmasculinos - 18–60+ years	Transfeminine: Estrogen (oral estradiol) with/without antiandrogens<br>Transmasculine: Testosterone (intramuscular or oral)	
Stoffer, Vries, Hannema, 2019(32)	Retrospective cohort study	Netherlands	62 transmasculine adolescents	transgender adolescents 17.2 years	Testosterone intramuscularly (IM), starting at 25 mg/m²/2 weeks → titrated to adult dose 125 mg/2 weeks; some transitioned to 250 mg/3–4 weeks or transdermal gel	1 year (range 5–33 months)
Liu et al., 2021(33)	Observational, retrospective cohort study	Northern Taiwan	110 65 trans masculine individuals<br>45 trans feminine individuals	Transgender individuals (ICD-10 F64.9) seeking gender-affirming hormone therapy<br>Mean age: 27.2 years	Trans masculine group: Testosterone cypionate Intramuscular injection \~166.6 mg every 2 weeks<br>Trans feminine group: Conjugated estrogen (oral, twice daily) ± cyproterone acetate (oral, daily)	4,5 anos
Martínez-Martín et al., 2023(34)	Retrospective cohort study	Las Palmas de Gran Canaria, Canary Islands, Spain	302<br>149 transgender women and 153 transgender men	Transgender women and transgender men - 19–22 years	Transgender men: Testosterone (83% parenteral, median dose: 1000 mg/3 months)	5 years
Lundberg et al., 2025(35)	Prospective longitudinal cohort study	Sweden	33 transgender men	33 transgender men<br>Early 20s at initiation, followed into 40s	Testosterone therapy: Primarily testosterone undecanoate<br>Dose: 1000 mg every 12 weeks (IM)	Median of 14 years (range: 10–20 years)

			Controls: 14 age-matched cisgender women		Some participants used testosterone gel intermittently	
Roy et. al., 2023(36)	Longitudinal observational metabolomics study	Colorado, United States	15 transgender and gender-diverse adolescents assigned female at birth	15 transgender men<br>Adolescents aged 13–16 years (15.0 ± 1.0 years) - 7 participants had received prior GnRHa (puberty blocker) treatment	Subcutaneous testosterone cypionate<br>Dose: Gradual escalation over 12 months<br>Route: Subcutaneous injection	1 year
Hamid et al., 2024(37)	Cross-sectional cohort study	Turkey	88<br>(44 transgender men and 44 cisgender women as controls)	Transgender men aged 20–50 who had:<br>Undergone hysterectomy and bilateral salpingo-oophorectomy (HBSO)<br>Been on testosterone therapy for >2 years<br>Control group: Healthy cisgender women matched by age<br>Mean age: 32.6 years	Testosterone therapy<br>Formulations: Testosterone undecanoate (n=11)<br>Testosterone isocaproate (n=33)<br>Dose: 250 mg intramuscularly<br>Duration of therapy: Mean 60.2 months (range 24–108 months)	Minimum 2 years<br>Mean duration: 5 years

Le, Pinkson, Trejo, Tripathy, 2024(38)	Retrospective observational study	United States (San Antonio, TX)	90 transgender adults<br>Transgender men: 36 Transgender women: 54	Trans men: 38 ± 2 years; Trans women: 46 ± 2 years	Trans men: Intramuscular testosterone cypionate	Trans men: 46 ± 5 months (~3.8 years)

eTable 2.B.: Summary of the 13 Included Studies Investigating Testosterone Therapy in Transgender Men (26–38).

**Author, Year**	**Cardiovascular Outcomes**	**Metabolic Outcomes**	**Mortality**
Gooren, Giltay and Bunck, 2008(26)	FtM: Slight increase in weight, ↓HDL, ↑triglycerides, minor changes in insulin sensitivity<br>Blood pressure unchanged; arterial stiffness not affected	FtM: ↓HDL, ↑CRP, ↑homocysteine, stable total/LDL cholesterol<br>↓insulin sensitivity in some studies Slight weight gain and fat redistribution	No increase in all-cause or cardiovascular mortality compared to Dutch general population
Meriggiola et. al.., 2008(27)	Events: No myocardial infarctions, strokes, or MACE reported Blood Pressure: Not significantly altered during treatment<br>Lipids<br>HDL: Decreased in all groups<br>Triglycerides & LDL: No significant change	↓ HDL cholesterol (statistically significant)<br>No changes in insulin sensitivity or glucose metabolism<br>↑ Hematocrit and hemoglobin as expected Bone markers remained stable across all groups	None reported<br>No adverse events led to discontinuation
Asscheman et al., 2011(28)	Ischemic heart disease, stroke, cardiovascular mortality Findings FtM Group: Only 1 death due to myocardial infarction after 42 years of testosterone	Direct metabolic biomarkers not reported in this paper.<br>However, previous reports by the same team showed testosterone was not associated with significant adverse metabolic changes.	FtM: 12/365 (3.4%) — similar to general population (SMR 1.12)<br>Testosterone: Appeared safe in FtM at standard replacement doses

Pelusi et al., 2014(29)	Blood pressure was not directly reported	HDL cholesterol decreased significantly ($p < 0.0005$)<br><br>LDL cholesterol increased significantly ($p = 0.001$)<br>No change in total cholesterol or triglycerides<br>Interpretation: No major cardiovascular events reported, but lipid changes indicate a less favorable profile<br><br>Glucose: Significant decrease across all groups ($p = 0.019$)<br>Insulin & HOMA-IR: No significant changes<br>Liver enzymes: Stable<br>Anthropometry: Weight and BMI increased slightly, fat mass decreased, lean mass increased in all groups	None during the 1-year study
Wierckx et al., 2014(30)	No cardiovascular events (e.g., MI, stroke, thromboembolism) reported during follow-up<br>Blood pressure changes: Not significant in trans men; mild decrease in trans women	Trans men:<br>↑ Hematocrit, hemoglobin, DHEAS, testosterone<br>↓ HDL cholesterol	No Deaths reported during the 12-month study period<br><br>No serious adverse events linked to hormone therapy observed

Getahun et al., 2018(31)	Transmasculine: No significant increase in cardiovascular risk	The study focuses on clinical events , not laboratory changes	Not detailed<br>No direct association with testosterone or estrogen use was identified.
Stoffer, Vries, Hannema, 2019(32)	No direct cardiovascular events (e.g., MI or stroke) reported; noted increase in systolic BP and hematocrit (up to >0.5 L/L in some)	↓ HDL, ↑ Hematocrit, Hemoglobin, DHEAS, Prolactin; BMI increased; BMD z-scores decreased and incomplete recovery	No deaths reported during the study
Liu et al., 2021(33)	Clinical CVD Events: Not directly reported<br>↑ BMI, LDL, hematocrit, hemoglobin in trans masculine group<br>↓ HDL in trans masculine group<br>These changes may reflect increased cardiovascular risk	Trans Masculine Group:<br>↑ BMI at all follow-ups<br>↑ LDL-C , ↓ HDL-C (significant at all visits)<br>↓ Insulin and HOMA-IR at 12–24 months<br>↑ Hemoglobin, Hematocrit, Creatinine at all visits<br>No significant changes in fasting glucose or HbA1c	None reported<br>No serious adverse events mentioned
Martínez-Martín et al., 2022(34)	No significant cardiovascular risks with testosterone	Weight, Fasting glucose, Creatinine, LDL cholesterol, Triglycerides, Systolic Blood Pressure (SBP)<br>No significant differences in lipid profile or glucose across groups (possibly masked by protocol-based lipid treatment)	None reported

Lundberg et al., 2023(35)	No major cardiovascular events (MI, stroke, thrombosis) reported<br>Blood pressure: Slight increase (~5 mmHg systolic)<br>Arterial stiffness and carotid intima-media thickness (CIMT): No significant changes over time	Lipids, glucose, insulin sensitivity (HOMA-IR), liver enzymes, hs-CRP<br>↑ Hematocrit and hemoglobin (within male reference range)<br>↓ HDL, ↑ LDL, but remained within acceptable range<br>Stable total cholesterol, glucose, HOMA-IR<br>↓ Fat mass and ↑ Lean mass (MRI confirmed)<br>Visceral fat remained low despite increased muscle mass	None reported<br>No serious adverse events during long-term follow-up
Roy et. al., 2023(36)	Outcomes monitored indirectly via biomarkers (no clinical events)<br>↑ Hematocrit (mild but statistically significant)<br>No major cardiovascular events (e.g., MI, stroke) reported<br>Metabolic shifts in RBCs suggest increased erythropoiesis and potential for altered oxygen offloading capacity	↑ Hematocrit<br>↓ Total cholesterol (non-significant)<br>↑ 2,3-DPG, glutathione, creatinine, urate<br>Altered glycolytic and amino acid metabolism in RBCs<br>Elemental shifts in sodium, magnesium, and calcium in RBCs over time	None reported<br>No serious adverse events related to testosterone therapy were observed

Continued

Hamid et al., 2024(37)	Measured markers: Internal and common carotid artery CIMT<br><br>Findings:<br>Significantly higher CIMT in transgender men vs. controls<br>64% had CIMT values above the 75th percentile vs. 16% in controls<br>CIMT positively correlated with testosterone duration, BP, and LDL<br>HDL inversely correlated with CIMT	Hemoglobin, hematocrit, HDL, LDL, triglycerides, HbA1c, HOMA-IR<br>↑ Body weight, BMI, LDL, triglycerides, hemoglobin, hematocrit<br>↓ HDL cholesterol<br>No significant change in HOMA-IR or fasting glucose	None reported<br>No major adverse cardiovascular events observed during study
Le, Pinkson, Trejo, Tripathy, 2024(38)	Trans men: Increase in diastolic blood pressure (72 → 76 mmHg, $p<0.05$)<br>No cardiovascular events (MI, stroke) reported	No significant changes in total cholesterol, LDL, HDL, or triglycerides in either group	Deaths Reported: None during the follow-up period<br>No adverse events related to hormone therapy were observed

Appendix 5: eFigure 2 - Cisgender Women (Randomized Controlled Trials)

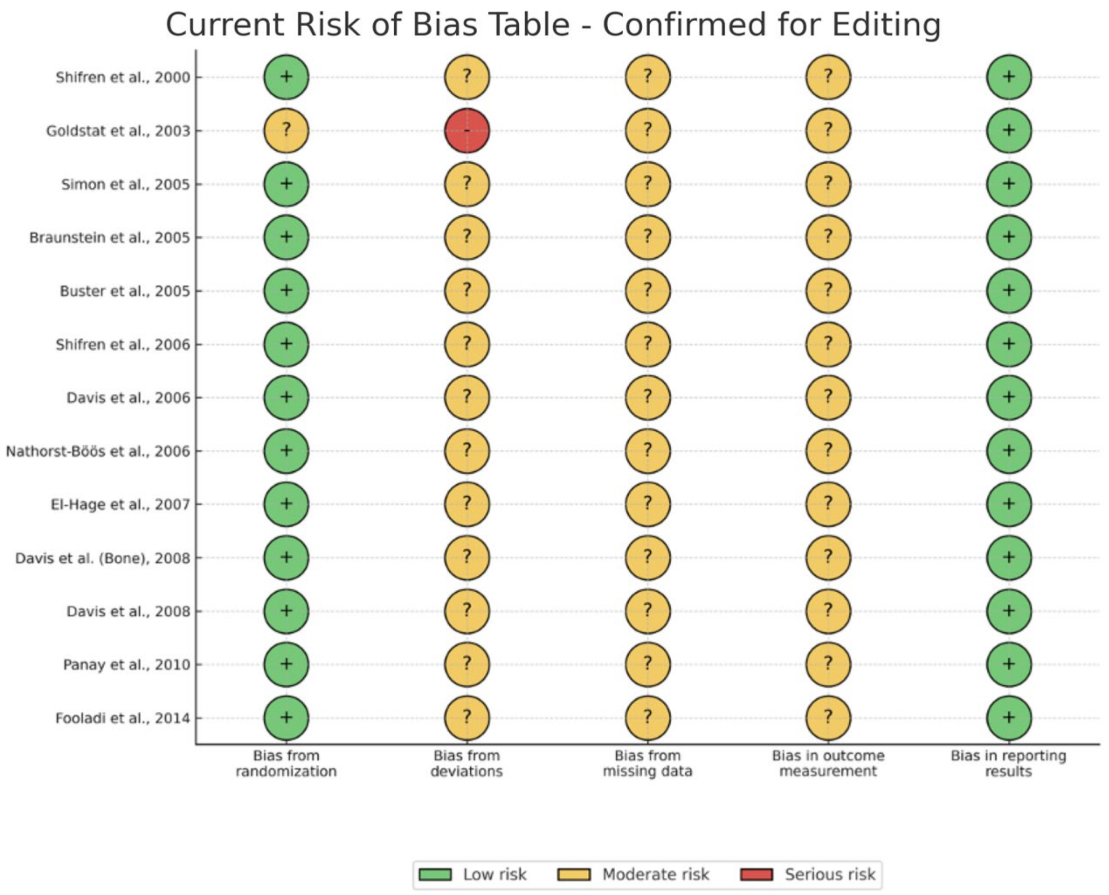

Appendix 6: eFigure 3 - Transgender Men (Observational Studies)

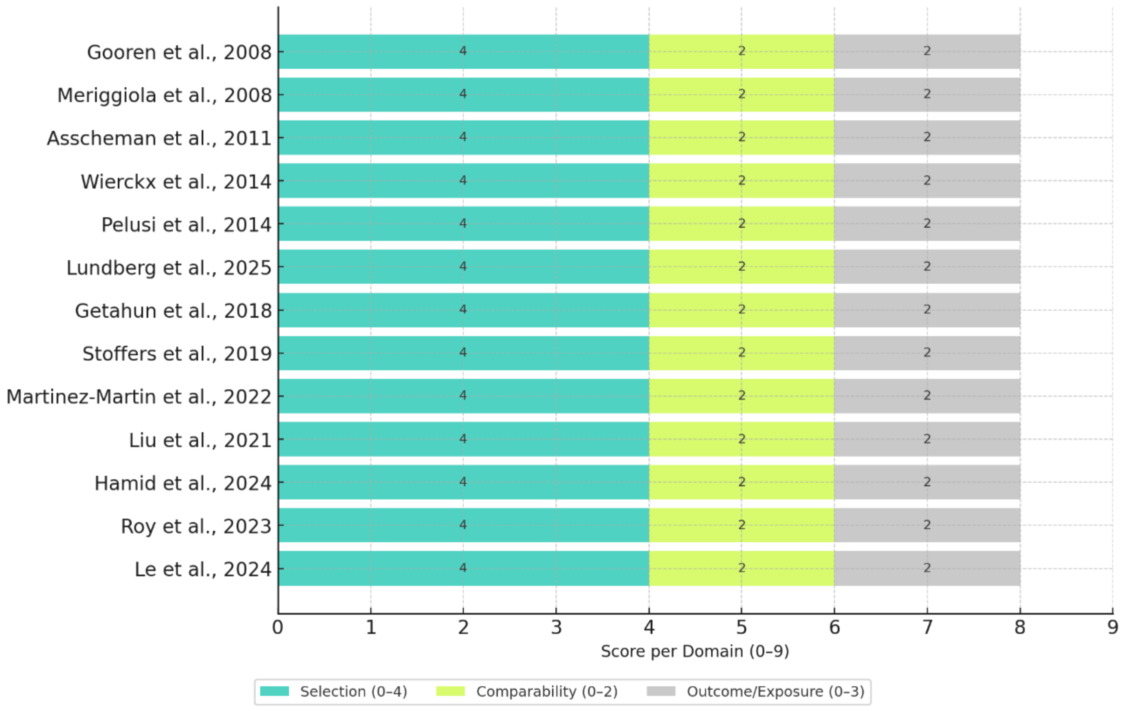

Appendix 7: eTable 3: Summary of Findings (SoF) Table: Testosterone Therapy for Cisgender Women

Outcomes	Risk with Placebo (Control)	Risk with Testosterone (Intervention)	Relative Effect (95% CI)	Absolute Effect (95% CI)	Participants (Studies)	(GRADE)
**Major Adverse Cardiovascular Events (MACE)[1]**	0 per 1,000	0 per 1,000	Not estimable	No difference	2,628 (13 RCTs)	⊕⊕⊕◯ Moderate[2]
**All-cause Mortality**	~0 per 1,000[3]	~0 per 1,000[3]	Not estimable	No difference	2,628 (13 RCTs)	⊕⊕⊕◯ Moderate[2]
**CLINICAL BENEFIT**						
**Increase in Satisfactory Sexual Events (per month)**	170 per 1,000 (baseline rate)	248 per 1,000	RR 1.48 (1.01 to 2.18)[4]	78 more per 1,000 (from 2 more to 201 more)	261 (1 RCT)[5]	⊕⊕⊕◯ Moderate[6]
**ADVERSE EFFECTS**						
**Mild Androgenic Effects (e.g., acne, hair growth)**	Low (e.g., ~50 per 1,000)	Significant increase	RR > 2.0 (estimated)[7]	Increase, generally mild and reversible	2,628 (13 RCTs)	⊕⊕⊕⊕ High

Table 3. Summary of Findings (SoF) table for cisgender women with HSDD treated with transdermal testosterone. Includes efficacy outcomes Explanatory Notes:

1. MACE (Major Adverse Cardiovascular Events): Includes myocardial infarction, stroke, and cardiovascular death.

2. Certainty of the Evidence (MACE / Mortality): Certainty starts as HIGH (data from RCTs) but is downgraded by one level to MODERATE due to very serious imprecision. The follow-up duration (maximum 52 weeks) is insufficient to capture long-latency cardiovascular events. Therefore, the absence of observed events reflects inadequate statistical power to assess risk, rather than confirmation of safety.

3. Mortality: The review text reports two non-treatment-related deaths (motor vehicle accidents), indicating that the overall mortality rate is extremely low and does not differ between groups.

4. Relative Effect (RR): A Relative Risk of 1.48 means that women in the testosterone group were 48% more likely to experience a satisfactory sexual event compared to the placebo group.

5. Efficacy Data: Data for this specific outcome were extracted from the Davis et al. (2008)(25) study, which is representative of the review's overall efficacy findings.

6. Certainty of the Evidence (Benefit): Certainty starts as HIGH (RCT) but is downgraded to MODERATE due to imprecision. The confidence interval of the relative effect (1.01 to 2.18) is very close to the line of no effect (1.0), which means the benefit, while present, might be small.

7. Androgenic Effects: The review consistently states that these effects were the most common adverse event, significantly more frequent in the testosterone group, but generally mild and reversible. The certainty is HIGH because this is a consistent and precise finding across multiple large RCTs.

Appendix 8: eTable 4: **Summary of Findings (SoF) Table: Testosterone Therapy for Transgender Men**

**Outcomes**	**Assumed Risk (Contextual Reference)*[1]**	**Risk with Testosterone**	**Relative Effect (95% CI)**	**Absolute Effect (95% CI)**	**Participants (Studies)**	**GRADE**
	**Cisgender male population reference (contextual)**	**Transgender Men**				
**Cardiovascular Mortality**	~2.05 per 1,000 person-years	~1.81 per 1,000 person-years	Uncertain; point estimate (RR 0.56) is likely biased by confounding.[2]	No clear signal of increased cardiovascular mortality observed within the limitations of low-certainty observational evidence.[2]	7,837 (7 observational studies)	⊕⊕◯◯ Low[3]
**Metabolic Changes**						
Unfavorable lipid changes (e.g., increased LDL, decreased HDL)	Not applicable (surrogate outcome)	Not applicable	Consistent findings of increased LDL and decreased HDL cholesterol.	Clinical significance of magnitude is uncertain.	3,419 (6 observational studies)	⊕◯◯◯ Very Low[4]
**Other Serious Risks**						
Suicide risk	Assumed 10 per 1,000 person-years[5]	~11 per 1,000 person-years	RR 1.08 (0.81 to 1.45)	0.8 more per 1,000 (from 1.9 fewer to 4.5 more)	5,040 (5 observational studies)	⊕⊕◯◯ Low[6]

Table 4. Summary of Findings (SoF) table for transgender men receiving gender-affirming testosterone therapy. Includes cardiovascular mortality, major adverse cardiovascular events (MACE), metabolic effects, and GRADE-based certainty assessment. Explanatory Notes:

* Cisgender male population data are provided solely for contextual interpretation and are not intended as formal comparators within the systematic review framework.

[1] Contextual reference: Baseline cardiovascular mortality estimates derived from cisgender male population surveillance data are provided solely for contextual interpretation. These data were not derived from the studies included in this systematic review and are not intended as formal comparators within the review framework.

[2] Cardiovascular mortality interpretation: The point estimate (RR 0.56) suggests a large apparent protective effect, which is biologically implausible and highly likely to reflect residual confounding, including healthy user bias. Accordingly, the evidence should not be interpreted as demonstrating protection. The most reliable **conclusion from this low-certainty observational evidence is the absence of a clear signal of increased cardiovascular mortality.**

[3] Certainty (cardiovascular mortality): Certainty starts as LOW due to observational study design and is not downgraded further, but the rating reflects a high risk of bias related to confounding and limited adjustment for baseline cardiovascular risk factors.

[4] Certainty (lipid changes): Certainty starts as LOW and is downgraded one level for serious indirectness, as lipid parameters are surrogate markers rather than clinical cardiovascular outcomes, and one level for serious risk of bias, resulting in VERY LOW certainty.

[5] Assumed suicide risk: Baseline risk is an illustrative assumption used to estimate absolute effects and does not represent a pooled estimate from included studies.

[6] Certainty (suicide risk): Certainty starts as LOW and is downgraded one level for serious imprecision due to wide confidence intervals encompassing both potential benefit and harm**.**

Appendix 9: eFigure 4 - Comparison of cardiovascular mortality across A comparative summary of testosterone exposure, follow-up duration, and cardiovascular mortality rates across groups

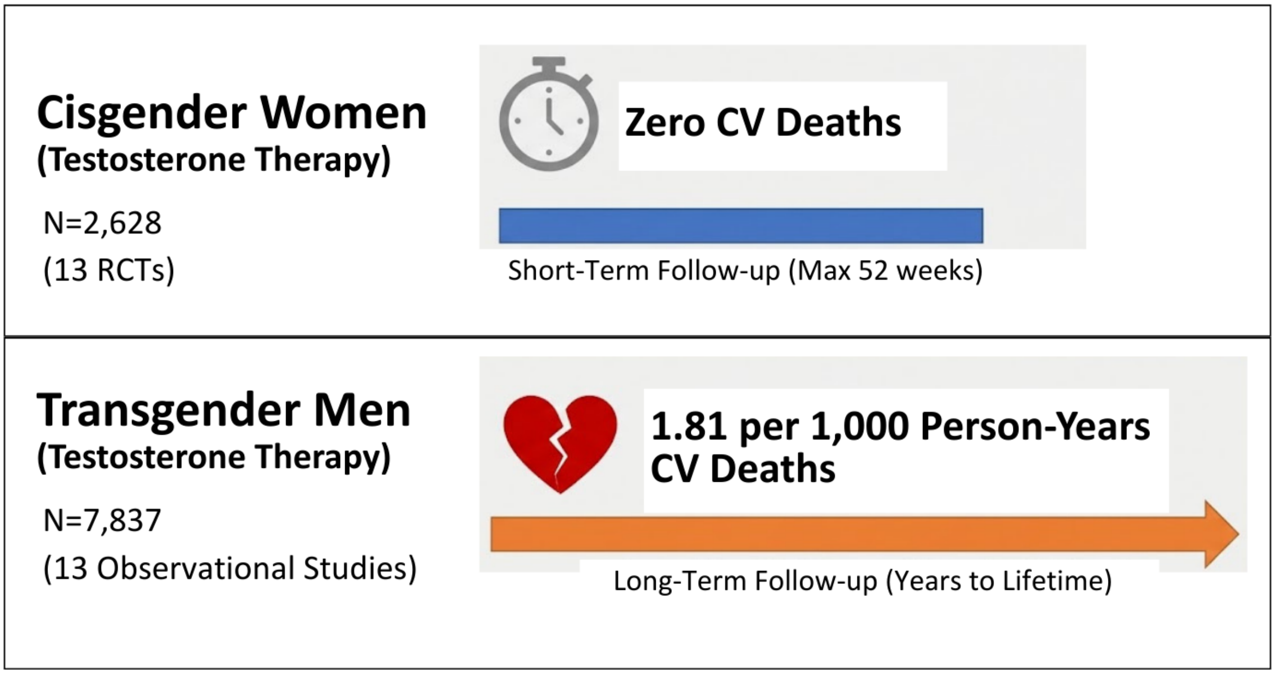

Note: The absence of reported cardiovascular deaths in the cisgender women group must be interpreted in the context of short-term follow-up (max 52 weeks) and does not imply long-term safety.

Figure 4. Visual Summary of Cardiovascular Mortality Outcomes Relative to Duration of Testosterone Exposure in the Included Studies. This figure illustrates the critical disparity in study duration and evidence types between populations. The top panel shows cisgender women receiving transdermal testosterone in RCTs. While zero cardiovascular deaths were reported (N=2,628), these trials were restricted to short-term follow-up (maximum 52 weeks). The bottom panel shows transgender men receiving masculinizing hormone therapy in observational cohorts, indicating a cardiovascular mortality rate of 1.81 deaths per 1,000 person-years over a significantly longer mean follow-up of 5.7 years (N=16,242).

Important Interpretation Note: The juxtaposition of these distinct populations is intended solely to highlight the gap in long-term data for cisgender women versus transgender men. The absence of observed cardiovascular events in the cisgender women group reflects insufficient statistical power and short exposure duration to detect rare, long-latency events, and must not be interpreted as confirmation of long-term cardiovascular safety. Direct comparison of risk rates between these groups is methodologically invalid due to fundamental heterogeneity in study designs (RCT vs. Observational), baseline population characteristics, routes of administration (transdermal stable levels vs. varied formulations including intramuscular peaks), and vastly different exposure timelines. CV = cardiovascular; RCT = randomized controlled trial.

Appendix 10: eTable 5: Summary of testosterone exposure, follow-up duration, and cardiovascular mortality rates across populations

Population	Testosterone Dose	Follow-up Duration	CV Deaths (n)	CV Mortality Rate (per 1,000 person-years)	Type of Data
Cisgender Women	~0.00003 mg/day (transdermal)	Up to 52 weeks	0	0	RCTs (short-term)
Transgender Men	14.29 mg/day (IM)	Mean 5.7 years	34	1.81	Observational cohorts
Cisgender Men	Endogenous physiological levels	Lifetime (epidemiological data)	—	2.05	Epidemiological surveillance (contextual reference)*

Comparison of testosterone type and dose, average or maximum follow-up duration, reported cardiovascular mortality rates, and the type of supporting evidence for each population group.

Footnote

* Cisgender male population data are provided solely for contextual interpretation and are not intended as formal comparators within the systematic review framework. These estimates were not derived from the studies included in this review.
